# COVID-19 Associated Thyroid Storm: A Case Report

**DOI:** 10.5811/cpcem.2021.5.52692

**Published:** 2021-08-27

**Authors:** Kevin Sullivan, Jana Helgeson, Andrew McGowan

**Affiliations:** Naval Medical Center San Diego, Department of Emergency Medicine, San Diego, California

**Keywords:** COVID-19, Thyroid storm, thyroiditis

## Abstract

**Introduction:**

The distinction between coronavirus disease 2019 (COVID-19) and thyroid storm can be extremely difficult to determine on clinical grounds alone as there is significant overlap between the signs and symptoms of each.

**Case report:**

We present a case of a patient with thyroid storm triggered by underlying COVID-19 infection.

**Conclusion:**

Infection with severe acute respiratory syndrome coronavirus 2 is linked to dysregulation of the thyroid gland through numerous mechanisms, although thyroid storm triggered by COVID-19 appears rare, with only a single case previously identified in the literature.

## INTRODUCTION

Infection with coronavirus disease 2019 (COVID-19) can lead to dysfunction of numerous organ systems, and associated thyroid disease is no exception.^1^–^3^ Both hypothyroidism and hyperthyroidism are documented complications of COVID-19 infection, but there is a paucity of data on the severity of hyperthyroidism in critically ill patients with COVID-19.^4^,^5^ There appears to be only a single case in the literature of overt thyroid storm triggered by severe acute respiratory syndrome coronavirus 2 (SARS-CoV-2) infection.^6^ We present a case of a critically ill patient with thyroid storm associated with COVID-19 infection.

## CASE REPORT

A 24-year-old female with a past medical history of Graves’ disease presented to the emergency department with one week of fever, cough, myalgias, and congestion. The patient’s vital signs included a heart rate of 133 beats per minute, blood pressure of 134/82 millimeters of mercury, respiratory rate of 24 breaths per minute, oxygen saturation of 98% on room air, and an oral temperature of 102.3° Fahrenheit. Her physical exam was notable for a thin, diaphoretic, and anxious-appearing female who was tremulous and had obvious exophthalmos.

Initial resuscitation included a 2-liter bolus of normal saline, 975 milligrams (mg) of oral acetaminophen, and 400 mg of oral ibuprofen. Antibiotics were considered but not administered given high initial suspicion for thyroid storm with viral trigger. A full complement of laboratory studies, including a complete blood count, basic metabolic panel, venous blood gas, blood cultures, thyroid function tests, urinalysis, and urine pregnancy test, were ordered. Diagnostic imaging included a portable chest radiograph (CXR). Computed tomography pulmonary angiography (CTPA) was considered, but the risks of worsening her potential thyrotoxicosis with an iodine-contrast load was thought to outweigh the benefits. Despite these interventions, the patient’s vital signs and clinical condition did not improve.

The patient’s labs showed an undetectable thyroid stimulating hormone and a free thyroxine of >7.770 nanograms per deciliter (ng/dL) (reference range 0.89–1.76 ng/dL) along with a positive SARS-CoV-2 polymerase chain reaction. The remainder of her laboratory results were unremarkable, and her CXR was without infiltrates. At this point, the patient was suspected to be in thyroid storm and had a Burch-Wartofsky score of 60. In consultation with endocrinology, the patient was started on 1 gram of propylthiouracil, 60 mg of propranolol, 100 mg of hydrocortisone, and 4 grams of cholestyramine. She had notable improvement in her symptoms and vital signs and was admitted to the intensive care unit (ICU) for further care. After 36 hours in the ICU, the patient was transferred to a medical-surgical floor and subsequently discharged home with endocrinology follow-up.

## DISCUSSION

There are multiple mechanisms by which thyroid dysfunction may occur in the setting of SARS-CoV-2 infection including lymphocytic proliferation of the thyroid and via a “cytokine storm” induced by the proinflammatory state of COVID-19.^7^ The thyroid gland has a relatively high concentration of angiotensin-converting enzyme 2 receptors, which facilitate entry of the virus into cells and may lead to direct thyroid infection.^8^ These mechanisms are especially relevant since even mild elevation in thyroid hormone in the setting of COVID-19 is linked to increased mortality.^4^

In the COVID-19 era, it is especially important for clinicians to maintain a high index of suspicion for thyroid storm in toxic-appearing patients. Many of the clinical features of COVID-19 infection including fever, fatigue, tachycardia, and diaphoresis overlap with those of thyroid storm making them difficult to distinguish.^9^,^10^ This has led some researchers to recommend routine thyroid function testing in all critically ill patients admitted for COVID-19.^8^ However, relevant studies in the workup of patients with COVID-19 often involve the use of iodine-containing contrast material. This further increases the risk of thyroid storm in predisposed individuals as described by the Jod-Basedow phenomenon, whereby an iodine load causes overproduction of thyroid hormone synthesis in patients with ineffective autoregulation.^11^

These patients may not manifest symptoms of hyperthyroidism until several weeks after the contrast load. This becomes especially problematic when attempting to exclude a diagnosis of pulmonary embolism (PE) in these patients. While infection with SARS-CoV-2 appears to be a risk factor for developing a PE, diagnosing this condition requires the use of a CTPA and thus a contrast load.^12^ It is also unclear what role D-dimer has in ruling out PE since D-dimer values tend to be elevated in these patients even in the absence of PE.^13^ Clinicians should strongly weigh the benefits and risks of testing for PE in patients with known thyroid disease who are also suspected of having COVID-19.

The treatment of thyrotoxicosis should not vary significantly in those with underlying COVID-19 compared to those with another trigger. Treatment of mild thyrotoxicosis in COVID-19 patients without underlying thyroid disease does not necessitate thionamides, and most of these patients will recover spontaneously.^4^ However, patients with thyroid storm should receive treatment with fluids, glucose repletion, thionamides, steroids, and beta blockade in conjunction with endocrinology consultation.^9^ Conveniently, treatment of critically ill COVID-19 patients with steroids may provide a mortality benefit and is also recommended in the treatment of thyroid storm.^14^

**CPC-EM Capsule**

What do we already know about this clinical entity?

*Infection with severe acute respiratory syndrome coronavirus 2 can trigger thyroid dysfunction leading to both hypothyroidism and hyperthyroidism*

What makes this presentation of disease reportable?

*The incidence of overt thyroid storm triggered by coronavirus disease 2019 (COVID-19) is rare, with only 2 cases reported in the literature.*

What is the major learning point?

*Clinicians must maintain a high index of suspicion for thyroid storm in patients presenting with COVID-19 as clinical features overlap.*

How might this improve emergency medicine practice?

*Consider ordering thyroid studies in all patients with severe COVID-19 infection.*

## CONCLUSION

Infection with SARS-CoV-2 has been linked with thyroid disease, but this is just the second case report of a patient with overt thyroid storm in the setting of COVID-19 infection. Clinicians should consider performing thyroid function testing in critically ill patients admitted with COVID-19, and they should be cautious of using iodine contrast in the diagnostic approach to these patients.
